# Design and fabrication of 4 double input NAND gate chip with excellent electrical and physical performances

**DOI:** 10.1038/s41598-025-14874-4

**Published:** 2025-08-11

**Authors:** Lijun Zhang, Wenqiang Dang, Yu Lu, Yongshun Wang

**Affiliations:** 1 School of Electronic and Information Engineering, Lanzhou Jiaotong University, Lanzhou, 730070 China; 2 School of Electronic and Information, Gansu Industry Polytechnic College, Tianshui, 741025 China; 3 Engineering Research Center, Ministry of Education on Integrated Circuit Packaging and Testing, Tianshui, 741001 China; 4 School of Electronic Information and Electrical Engineering, Tianshui Normal University, Tianshui, 741001 China; 5 Tianshui Tianguang Semiconductor Co., Ltd., Tianshui, 741000 China

**Keywords:** DC characteristics, Dynamical characteristics, ESD protection, Anti-latch-up, Radiation re-sistance, Electrical and electronic engineering, Computer science

## Abstract

In order to improve the performances of 4 two-input NAND so that it can be better used in the aerospace application field and in harsh environments, 4 two-input NAND gate chips were designed and successfully fabricated in this paper, based on 1.2 ${\upmu }$m P-well SPDM CMOS technological processes. The N transistors were fabricated in P-well and connected to GND via $P^+$-well. The metal wires of $V_{CC}$ use $N^+$ substrate to contact $V_{CC}$, to increase its anti-locking capability. An ESD protection circuit was designed at the input terminals, prohibiting the terminal of IC chip from being damaged by ESD stress. The performance of the device is superior to that of other similar products in the world.

## Introduction

CMOS 4 two-input NAND gate IC has many advantages such as low static power dissipation, high noise margin, compatible with standard LSTTL IC, and a high driving load capacity for 10 LSTTL^1–4^. When all the input terminals are at a high level, the output is at a low level only. All the inputs are clamped to $V_{CC}$ and to the ground voltage for a low level by the internal diodes to avoid electrostatic discharge damage. In order to improve the anti-static performance, the ESD protection structure was designed in this paper. The protection ring was designed to reduce latch-up probability in the cutoff region. The chips were designed based on 5 V 1.2 ${\upmu }$m P-well SPDM CMOS process. The N transistors were placed in the P-well, which was connected to GND through $P^+$-well. The metal wire layout uses as much as possible $N^+$ substrate contact connected to $V_{CC}$ to further improve the anti-latch-up capability.

Since the 4 two-Input NAND Gate IC consists of four identical parts, the layout was designed symmetrically both in horizontal and vertical directions. The maps of the active region and polycrystalline silicon were drawn based on the structure and geometric dimensions of MOSFETs^5–11^. In order to decrease the chip area and to make the IC layout clear and tidy, the strips of polycrystalline silicon were tidily arranged as densely as possible according to the minimum process principle. The protection rings were designed and fabricated for the terminal devices.

## Results

### Design technical specifications (TA = 25 $^{\circ }$C)

- Power voltage: 2 V - 6 V
  High level output voltage ($V_{OH}$):
  $$\ge$$ 1.9 V ($$I_{OH}$$ = -20 $${\upmu }$$A, $$V_{CC}$$ = 2.0 V)
  $$\ge$$ 4.4 V ($$I_{OH}$$ = -20 $${\upmu }$$A, $$V_{CC}$$ = 4.5 V)
  $$\ge$$ 3.98 V ($$I_{OH}$$ = -4 mA, $$V_{CC}$$ = 4.5 V)
  $$\ge$$ 5.9 V ($$I_{OH}$$ = -20 $${\upmu }$$A, $$V_{CC}$$ = 6.0 V)
  $$\ge$$ 5.48 V ($$I_{OH}$$ = -5.2 mA, $$V_{CC}$$ = 6.0 V)
  Low level of output voltage ($V_{OL}$):
  $$\le$$ 0.1 V ($$I_{OL}$$ = 20 $${\upmu }$$A, $$V_{CC}$$ = 2.0 V, 4.5 V or 6.0 V)
  $$\le$$ 0.26 V ($$I_{OL}$$ = 4 mA, $$V_{CC}$$ = 4.5 V)
  $$\le$$ 0.26 V ($$I_{OL}$$ = 5.2 mA, $$V_{CC}$$ = 6.0 V)
- Power current:
  $$\le$$ 2 $${\upmu }$$A
- Transmission delay time ($t_{PLH} /t_{PHL}$):
  $$\le$$ 90 ns ($$C_L$$ = 50 $$\times$$ (1±10%) pF, $$V_{CC}$$ = 2.0 V)
  $$\le$$ 18 ns ($$C_L$$ = 50 $$\times$$ (1±10%) pF, $$V_{CC}$$ = 4.5 V)
  $$\le$$ 15 ns ($$C_L$$ = 50 $$\times$$ (1±10%) pF, $$V_{CC}$$ = 6.0 V)
- Conversion time ($$t_{TLH} /t_{THL}$$):
  $$\le$$ 75 ns ($$C_L$$ = 50 $$\times$$ (1±10%) pF, $$V_{CC}$$ = 2.0 V)
  $$\le$$ 15 ns ($$C_L$$ = 50 $$\times$$ (1±10%) pF, $$V_{CC}$$ = 4.5 V)
  $$\le$$ 13 ns ($$C_L$$ = 50 $$\times$$ (1±10%) pF, $$V_{CC}$$ = 6.0 V)
- Operation temperature:
  -55 $$^{\circ }$$C to +125 $$^{\circ }$$C

### Circuit structure design

**Fig. 1:**
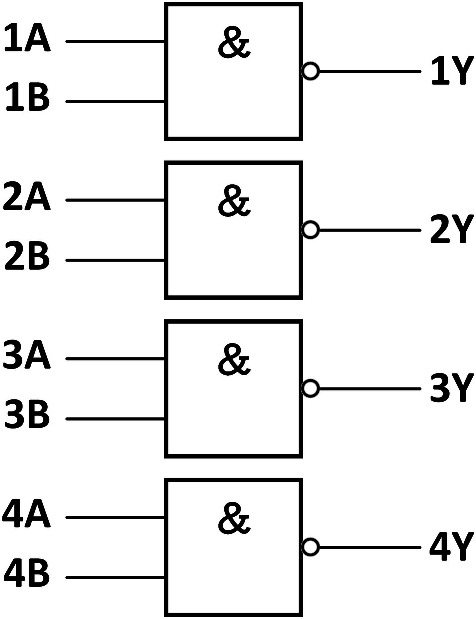
Four two-input NAND gate.

Four two-input NAND gates are integrated on a chip. Each NAND gate has two inputs and one output. When all of its inputs are at a high level, the output is at a low level; otherwise, it is at a high level. It can be written as a mathematical logical expression Y = $\overline{A \cdot B}$, and its logic diagram was shown in Fig. 1.

#### Input buffer module

**Fig. 2:**
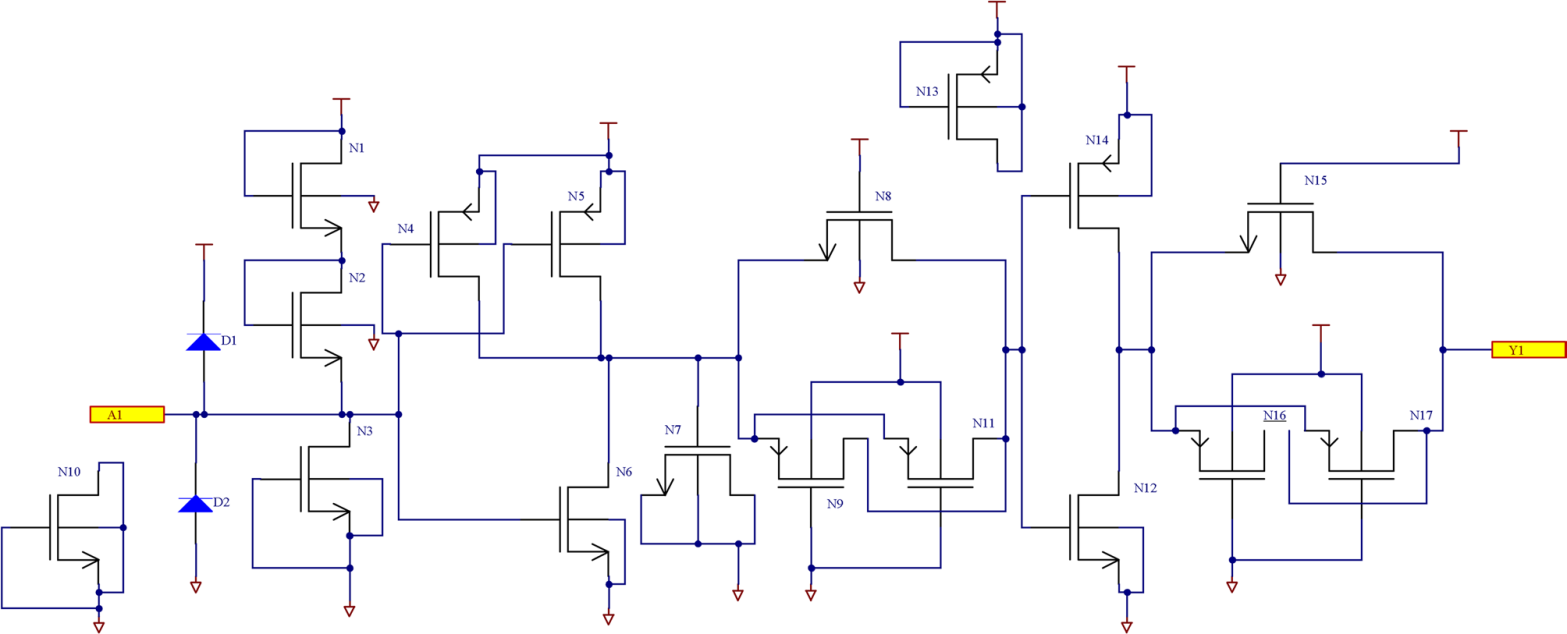
Schematic diagram of input buffer module.

The input buffer module consists of 2 inverters and 2 normally-on transmission gates. Its output voltage is identical to the input voltage, and it is used to quickly transfer signals and stabilize them. The transmission gate is designed with NMOS and PMOS transistors, where the drain and source are connected together as input and output terminals, respectively, as shown in Fig. 2.

The transmission gate offers several advantages, including:

- No voltage threshold loss:
  Due to the complementary operation of NMOS and PMOS transistors, the transmission gate can transfer signals without threshold voltage drop.
- No substrate bias effect:
  The design avoids the body effect, ensuring stable performance.
- Efficient low-level signal transmission:
  It can efficiently transmit low-level signals with high fidelity.
- High charging current:
  The parallel structure of NMOS and PMOS provides a high charging current for fast signal transfer.
- Full-swing signal transfer:

Since one of the two devices is always conductive, the transmission gate can transfer full-swing signals.

#### Intermediate and output driving stages

**Fig. 3:**
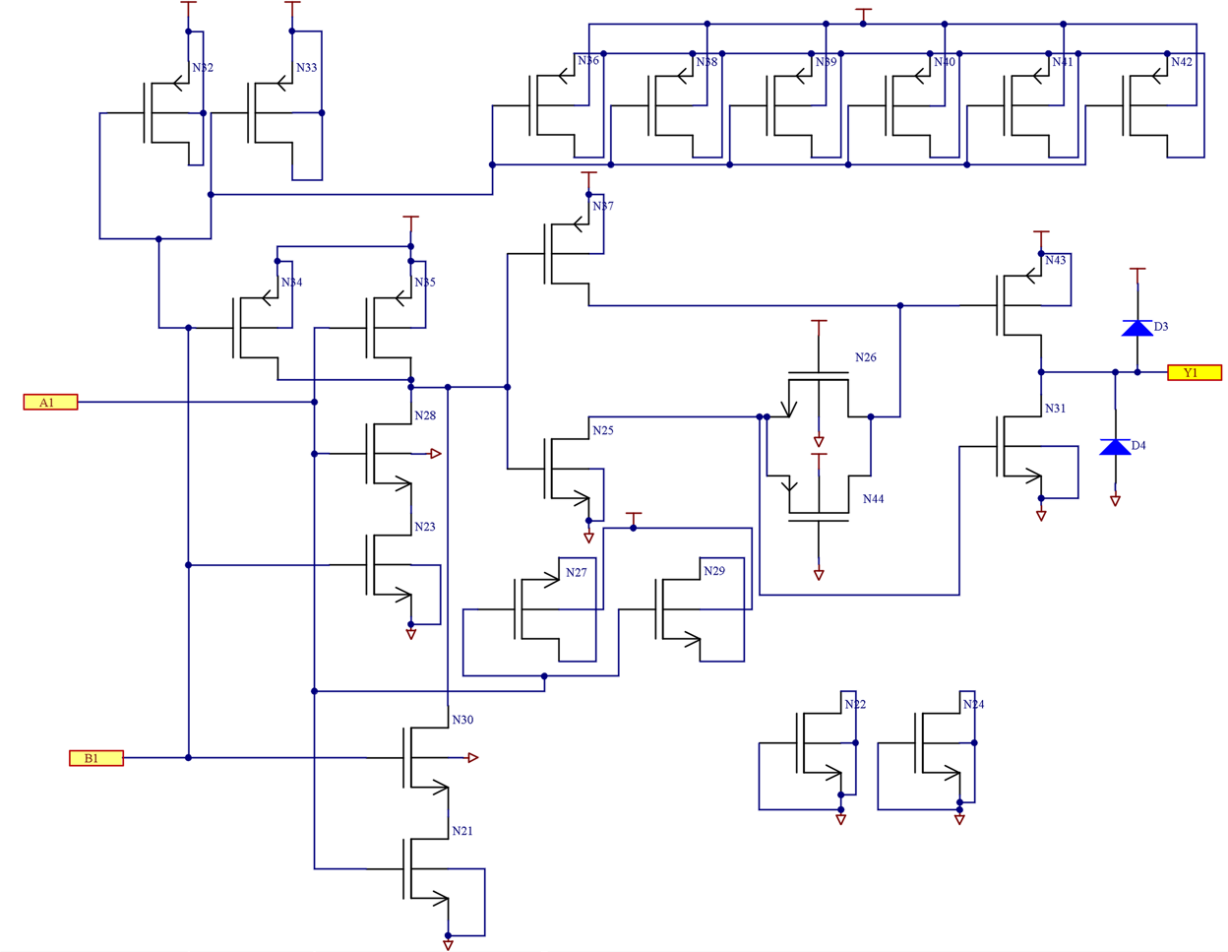
The circuit principle diagram of the intermediate and output driving stage.

**Fig. 4:**
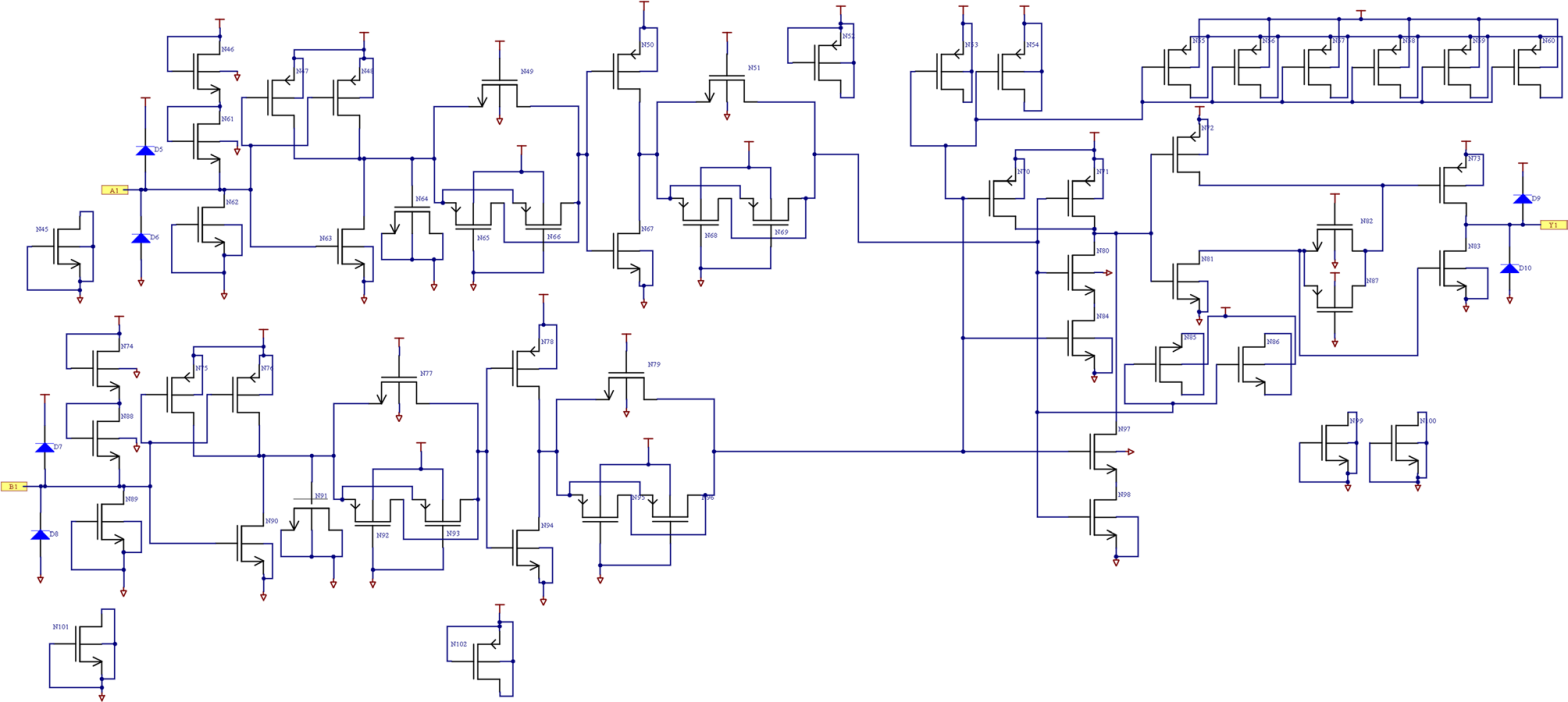
Schematic diagram (1/4) of designed 4 two-NAND gate.

The circuit principle diagram of the intermediate and output driving stage is shown in Fig. 3. A NAND gate is used as an intermediate stage, and the output driver is composed of two inverters and one transmission gate. The output voltage level follows the changes in the input level. One-fourth of the schematic diagram of the designed 4 two-input NAND gate is illustrated in Fig. 4.

### Simulation for functions and electrical parameters

According to the technical specifications, the static and dynamical characteristics were simulated at three temperatures TA = 25 °C, TA = − 55 °C and TA = 125 °C. The simulation results can well satisfy the application requirements and design specifications.

#### Function simulation

**Fig. 5:**
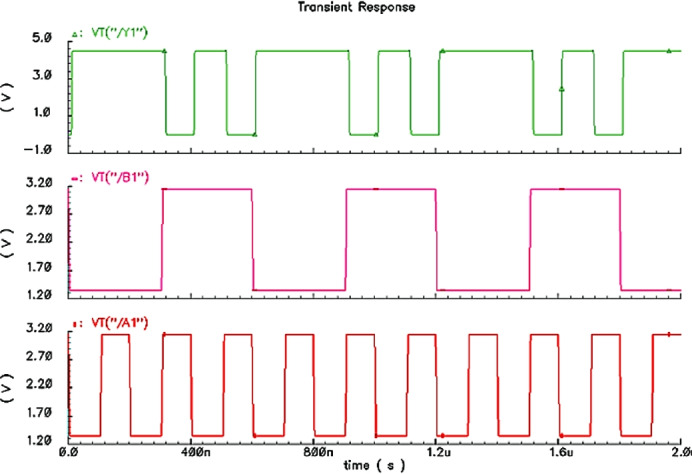
Function simulation waveforms.

As shown in Fig. 5, the simulation results indicate the designed circuit has correct NAND gate characteristics with excellent performance for three temperatures.

#### Steady state characteristic simulation

The important DC parameters including $V_{OH}$, $V_{OL}$, $I_I$ and $I_{CC}$, were simulated for different temperatures TA = 25 $^{\circ }$C, TA = -55 $^{\circ }$C and TA = 125 $^{\circ }$C. The simulated results meet the post-service standard, as listed in Table 1.

**Table 1:** Steady state characteristic simulation.

Parameter	Conditions	V_CC_	Specifications	25 °C		Specifications	−55 °C	125 °C
				Typical	Simulation results		Simulation results	
Output high level ($V_{OH}$ )	$V_{IA}$ = $V_{IH}$ $V_{IB}$ = $V_{pulse}$	2V	$\ge$ 1.9V	1.998V	1.99817V	$\ge$ 1.9 V	1.99854 V	1.99784 V
		4.5 V	$\ge$ 4.4 V	4.499 V	4.49923 V	$\ge$ 4.4 V	4.49941 V	4.49903 V
		6 V	$\ge$ 5.9 V	5.999 V	5.99931 V	$\ge$ 5.9 V	5.9995 V	5.99927 V
	$V_{IA}$ = $V_{IH}$ $I_{OH}$ = -20 ${\upmu }$A $V_{IB}$ = $V_{pulse}$ $I_{OH}$ = -4 mA	4.5 V	$\ge$ 3.98 V	4.3 V	4.34224 V	$\ge$ 3.7 V	4.38134 V	4.30289 V
	$V_{IA}$ = $V_{IH}$ $V_{IB}$ = $V_{pulse}$ $I_{OH}$ = -5.2 mA	6 V	$\ge$ 5.48 V	5.8 V	5.83025 V	$\ge$ 5.2 V	5.87024 V	5.79254 V
Output low level ($V_{OL}$ )	$V_{IA}$ = $V_{IH}$ $V_{IB}$ = $V_{IH}$	2 V	$\le$ 0.1 V	0.002 V	1.32206 mV	$\le$ 0.1 V	824.83 ${\upmu }$V	2.1128 mV
		4.5V	$\le$ 0.1 V	0.001 V	590.369 ${\upmu }$V	$\le$ 0.1 V	397.17 ${\upmu }$V	912.054 ${\upmu }$V
		6 V	$\le$ 0.1 V	0.001 V	503.78 ${\upmu }$V	$\le$ 0.1 V	351.108 ${\upmu }$V	744.141 ${\upmu }$V
	$V_{IA}$ = $V_{IH}$ $I_{OH}$ = 20 ${\upmu }$A $V_{IB}$ = $V_{IH}$ $I_{OH}$ = 4 mA	4.5 V	$\le$ 0.26 V	0.17 V	121.136 mV	$\le$ 0.4 V	80.913 mV	189.294 mV
	$V_{IA}$ = $V_{IH}$ $V_{IB}$ = $V_{IH}$ $I_{OH}$ = 5.2 mA	6 V	$\le$ 0.26 V	0.15 V	134.357 mV	$\le$ 0.4 V	93.1202 mV	200.127 mV
Input current ($I_{I}$ )	$V_{I}$ = $V_{CC}$	6 V	$\le$ ±100 nA	±0.1 nA	− 180 nA	$\le$ ±1000 nA	− 17.9997 pA	− 180.158 nA
	$V_{I}$ = 0 V				10.5484 pA		10.3103 pA	418.899 pA
Power current ($I_{CC}$ )	$V_{I}$ = $V_{CC}$	6 V	$\le$ 2.0 ${\upmu }$A	/	659.995 nA	$\le$ 40 ${\upmu }$A	66.0189 pA	661.264 nA
	$V_{I}$ = 0 V				75.1929 pA		74.6816 pA	1.47074 nA

### Dynamical characteristic simulation

The dynamical parameters such as transmission delay times ($t_{PHL}$, $t_{PLH}$) and output conversion times ($t_{THL}$, $t_{TLH}$) were simulated at the different temperatures TA = -55 $^{\circ }$C, TA = 25 $^{\circ }$C and TA = 125 $^{\circ }$C, respectively. The simulated results satisfy the application requirements, as listed in Table 2.

**Table 2:** Dynamical characteristic simulation.

Parameter	Symbol	Conditions		25 °C		Specifications	− 55 °C	125 °C
				Specification	Simulation results		Simulation results	
Transmission delay time $V_{A1}$ , $V_{B1}$ pulse	$t_{PLH}$	A to Y	$V_{CC}$ = 2.0 V	$\le$ 90 ns	42.6816 ns	$\le$ 134 ns	39.6542 ns	46.5763 ns
		B to Y			42.705 ns		39.6571 ns	46.5685 ns
	$t_{PHL}$	A toY			53.9354 ns		43.9511 ns	64.7179 ns
		B toY			53.9392 ns		43.9104 ns	64.7501 ns
	$t_{PLH}$	A to Y	$V_{CC}$ = 4.5V	$\le$ 18 ns	12.4692 ns	$\le$ 27 ns	9.73348 ns	19.73 ns
		B to Y			12.4649 ns		9.74114 ns	19.7328 ns
	$t_{PHL}$	A to Y			16.3921 ns		12.0944 ns	22.7842 ns
		B to Y			16.3812 ns		12.0967 ns	22.7804 ns
	$t_{PLH}$	A to Y	$V_{CC}$ = 6.0V	$\le$ 15 ns	9.89179 ns	$\le$ 23 ns	7.90145 ns	12.3725 ns
		B to Y			9.8948 ns		7.89851 ns	12.3647 ns
	$t_{PHL}$	A to Y			12.9485 ns		9.67343 ns	17.413 ns
					12.9427 ns		9.68032 ns	17.4296 ns
Conversion time $V_{A1}$ , $V_{B1}$ Pulse	$t_{TLH}$	Y	$V_{CC}$ = 2.0 V	$\le$ 75 ns	19.6105 ns	$\le$ 110 ns	16.712 ns	22.3156 ns
	$t_{THL}$				18.3071 ns		13.342 ns	25.4582 ns
	$t_{TLH}$		$V_{CC}$ = 4.5 V	$\le$ 15 ns	6.80038 ns	$\le$ 22 ns	5.22523 ns	8.69869 ns
	$t_{THL}$				7.77215 ns		5.74331 ns	11.0014 ns
	$t_{TLH}$		$V_{CC}$ = 6.0 V	$\le$ 13 ns	5.47886 ns	$\le$ 19 ns	4.30546 ns	6.93662 ns
	$t_{THL}$				6.60259 ns		4.92112 ns	9.00255 ns

### Threshold simulation (TA = 25 $^{\circ }$C)

**Fig. 6:**
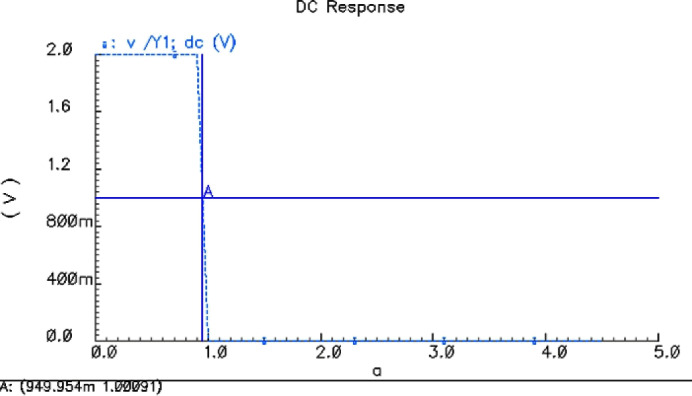
Threshold simulation results for VCC = 2.0 V.

**Fig. 7:**
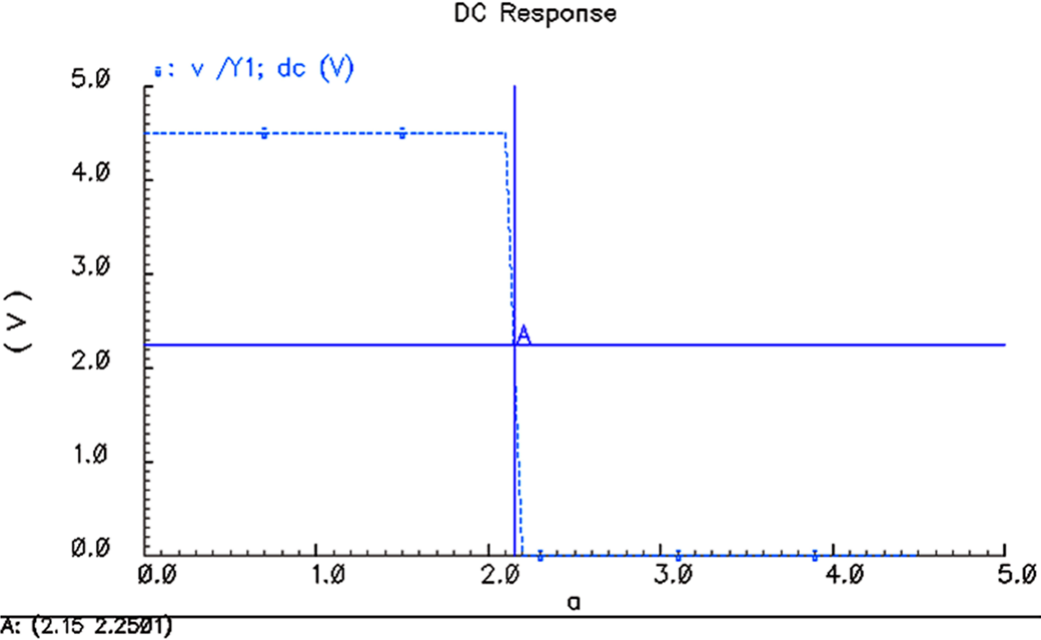
Threshold simulation results for VCC = 4.5 V.

**Fig. 8:**
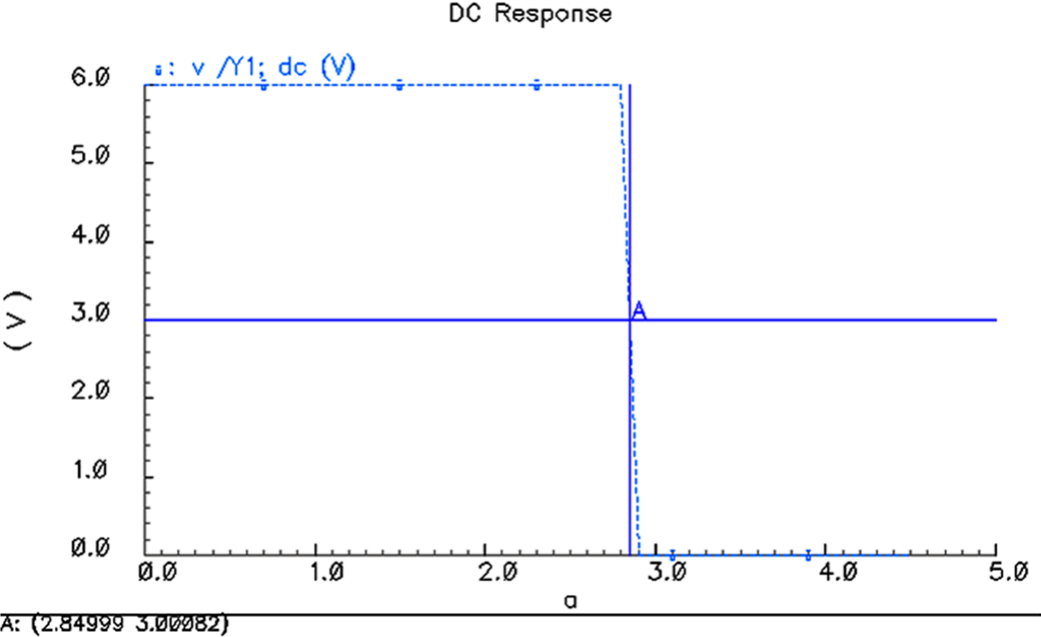
Threshold simulation results for VCC = 6.0 V.

- Voltage transmission characteristics for 2 V
  The simulation results for $V_{CC}$ = 2.0 V are shown in Fig. 6, and the inversion voltage of 4 two-NAND gate is 949.954 mV.
- Voltage transmission characteristics for 4.5 V
  The simulation results for $V_{CC}$ = 4.5 V are shown in Fig. 7, and the inversion voltage of 4 two-NAND gate is 2.15 V.
- Voltage transmission characteristics for 6 V
  The simulation results for $V_{CC}$ = 6.0 V are shown in Fig. 8, and the inversion voltage of 4 two-NAND gate is 2.84999 V.

### Layout design

The 4 two-Input NAND Gate was constructed with four identical parts, and the layout was distributed symmetrically both in horizontal and vertical directions. The active region and polysilicon were drawn as neatly and intensively as possible according to the geometric structure and minimum process sizes, to further decrease the chip area. The protection rings were designed around the terminal device.

#### Layout design of unit modules

**Fig. 9:**
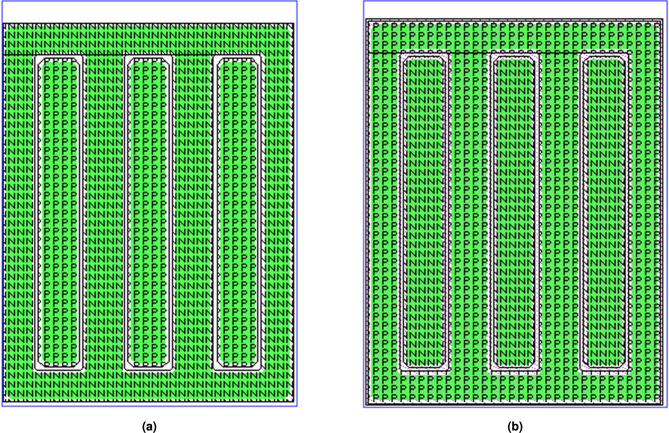
ESD-protection diode layout: (**a**) Diodes at input and output terminals, which consist of $P^+$ and N-substrate; (**b**) A diode between $V_{CC}$ and GND, which consists of $N^+$ and P-well.

**Fig. 10:**
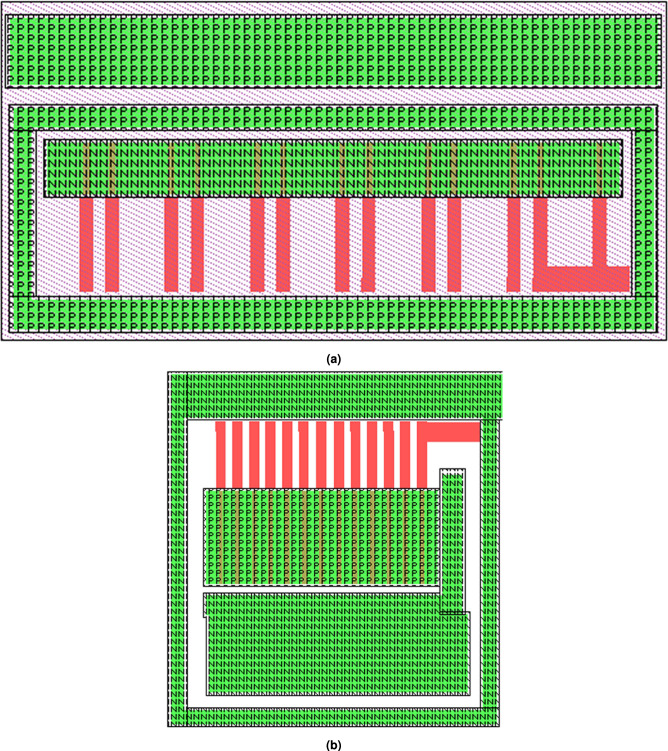
Structure diagram of anti-latch-up: (**a**) NMOS transistor surrounded with a $P^+$ active region, (**b**) PMOS transistor surrounded with an $N^+$ active region.

**Fig. 11:**
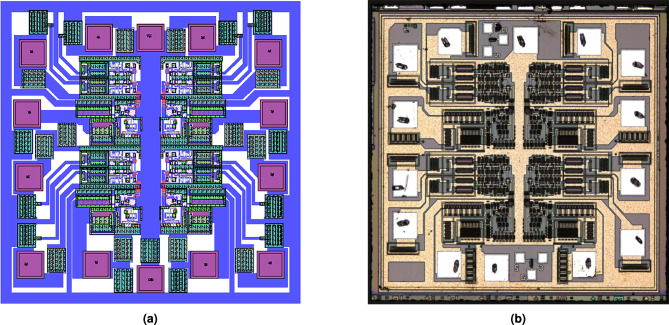
Layout of designed devices (**a**) Profile of layout, (**b**) Actual layout of Chip.

- Improvements in Antistatic ability
  As shown in Fig. 9, a diode-based ESD protection structure was designed due to its advantages, including stable performance, ease of process control, and low power dissipation during the discharge period. The structure consists of diodes formed by $P^+$ regions and the N-type substrate, connecting each input and output terminal to $V_{CC}$ with a circular junction design. Additionally, a diode formed by an $N^+$ region and a P-well is added between $V_{CC}$ and GND, as illustrated in Fig. 9b.
- Design of anti-latch-up
  The latch-up effect, also called lock-in effect, is inherent in CMOS integrated circuit. Extrinsic noise voltage at the input or output terminals can activate the parasitic bipolar NPN and PNP transistors in CMOS ICs as a thyristor, generating a damaging current from $V_{CC}$ to GND. In order to diminish the latch-up effects effectively, a protection ring structure was designed in this 4 two-Input NAND Gate. To improve anti-latch-up capability, the NMOS transistors were surrounded with a $P^+$ active region as shown in Fig. 10a, PMOS with an $N^+$ active region, as shown in Fig. 10b. The overview layout of it is shown in Fig. 11.
- Reliability consideration
  In order to improve the antistatic capability, an ESD protection structure was designed for all input and output terminals. The anti-latch-up structure was designed and a protection ring surrounds MOS transistor. The NMOS transistor was surrounded with a $P^+$ protection ring, whereas the PMOS was surrounded with an $N^+$ protection ring, increasing the anti-latch-up performance. At the same time, the distances from well boundary to source and drain regions of MOS transistors, as well as the space between NMOS and PMOS transistors, were appropriately increased to further improve the anti-latch-up capability. In order to decrease the occurrence probability of pinholes, the area covered by aluminum strip should be minimized, and the length of the aluminum (Al) strip should also be as short as possible. Using a short Al strip can further reduce the area and increase the transmission speed. The wide metal strips were replaced with ones having many trough-shaped profiles. The wide metal strip with rectangular windows spaced at a certain distance can effectively prevent or eliminate the destruction of the passivation layer caused by metal heat expansion and prevent impurities from penetrating into the active regions of chips. When the current-carrying capability of aluminum strips is much higher than the practical operation current of devices, electric migration phenomena can be avoided, ensuring operation reliability.

#### Layout verification

In order to make the design conform to technological process standard, the layout design rule was checked using DRC files. To ensure the layout to be consistent with the circuit, it was comparatively checked with the circuit using LVS files, the layouts of the chip were identical with its circuit.

### Protection circuit design

An ESD protection circuit was designed at the device terminals to prevent the devices from being damaged. The charges induced by static induction cannot be leaked when the gate electrode is in a floating state because of the gate insulating resistors of CMOS devices^1,12–15^. Since the gate oxide film of CMOS devices is very thin, when the electric field induced by the static-induction charges in the gate oxide layer is higher than the breakdown field intensity of the oxide film, the gate may be permanently damaged. Usually, when the intensity of the electric field generated by the accumulated charges on the gates is higher than 10$^7$ V/cm, the thin gate oxide film may break down. For the purpose of protecting IC chip terminals from ESD stress-induced damage, an ESD protection circuit was designed at the input terminals. In designing the ESD protection device, multiple factors such as coupling, decoupling, buffering, ballasting, triggering, and shunting need to be considered. The main design objective of the ESD protection device is to ensure that the working circuit is not used as a discharging path and thus is not damaged. When ESD occurs between any two terminals, a suitable low-resistance bypass is required. This bypass can not only absorb the ESD current but also clamp the operating voltage to prevent over-voltage. The channels of the ESD protection circuit possess excellent stability and can respond rapidly when ESD occurs without interfering with the normal operation of the chips.

#### Protection circuit composed of diodes

**Fig. 12:**
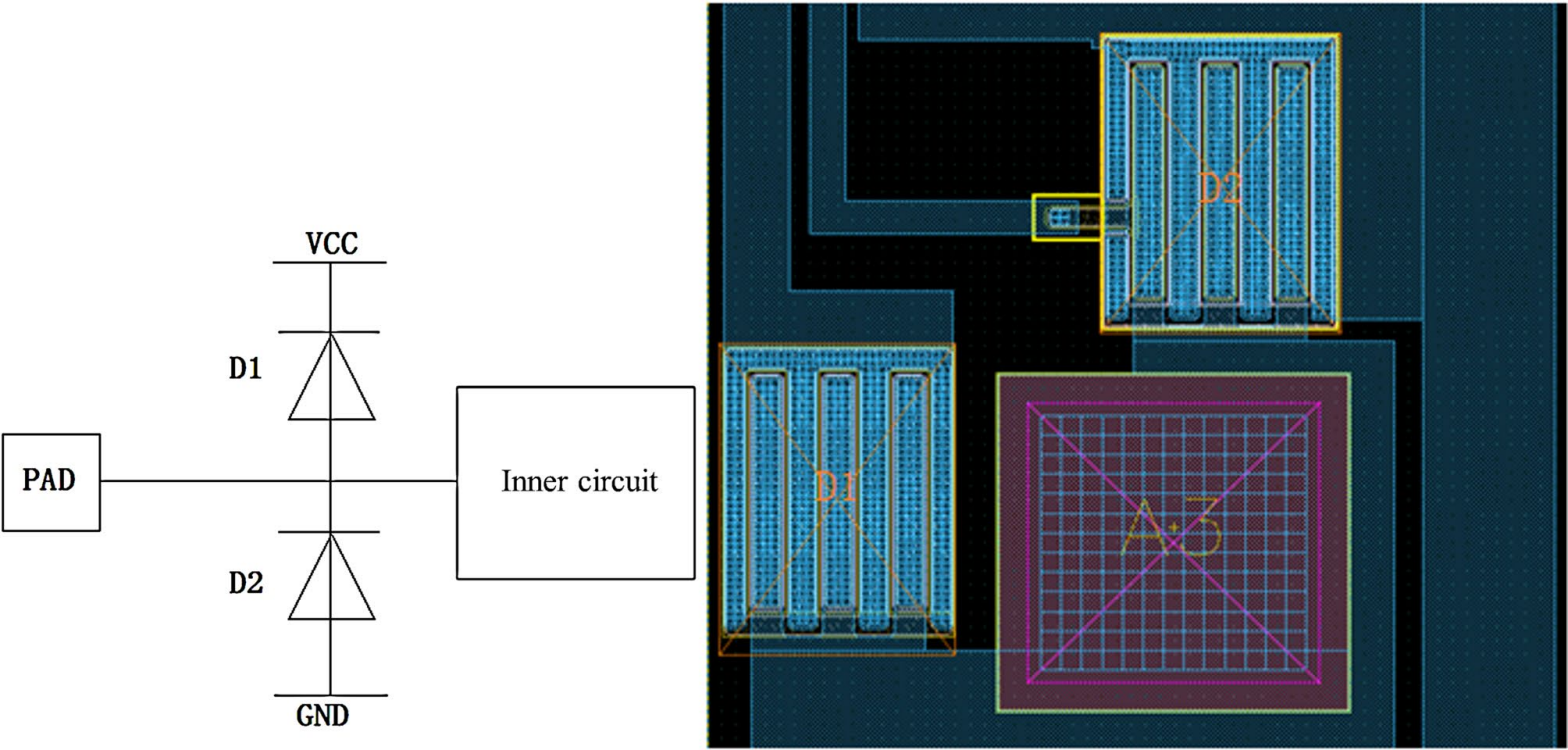
Discharging protection circuit and its layout at OE and DIR terminals.

The diode D1, which is connected to $V_{CC}$ consisting of $P^+$ and N substrate, and D2, which is connected to GND composed of $N^+$ and P-well, were designed with circular junctions, increasing their breakdown voltage. As shown in Fig. 12, when the voltage input from PAD is higher than the power supply voltage $V_{CC}$, D1 turns on and the input voltage is clamped to $V_{CC}$+$V_d$ (Forward Voltage Drop $V_d$); when the input voltage is lower than GND, the diode D2 turns on and the input voltage is clamped to -$V_d$. Therefore, the applied input voltage is clamped to the range of -$V_d$ to $V_{CC}$+$V_d$, which is a safe voltage for this product.

#### ESD protection structure between VCC and GND

**Fig. 13:**
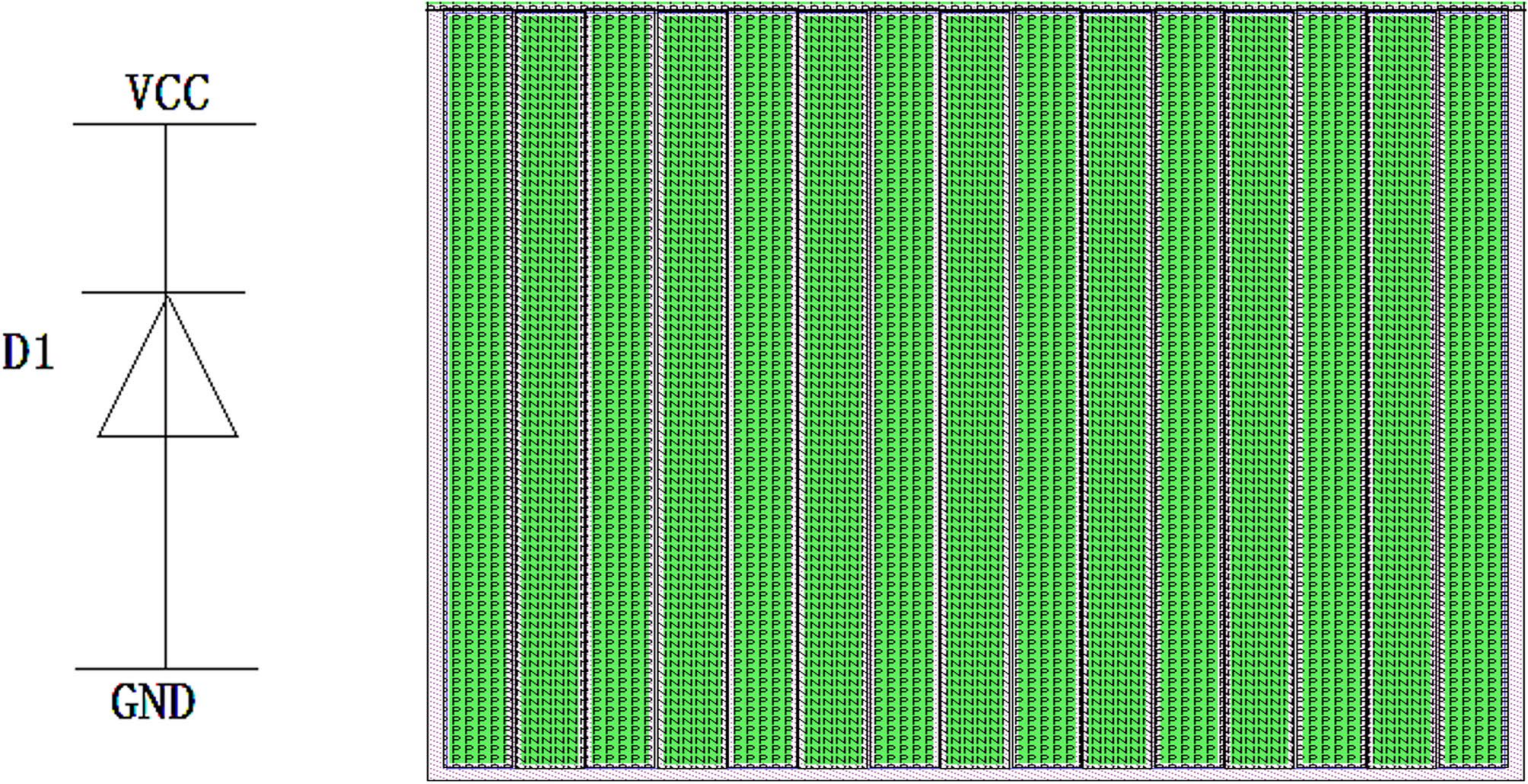
Circuit diagram and Layout of ESD protection circuit between $V_{CC}$ and GND.

The inversely biased PN junctions with enough area between power supply and GND in large-scale IC can serve as an ESD protection circuit. Whereas for the small- or medium-scale IC, it is necessary to have an inversely biased diode between power supply and GND as a discharging channel. The protection circuit consists of many parallel diodes composed of $N^+$ connected to $V_{CC}$ and P-well which is connected to GND through $P^+$ for this product, as shown in Fig. 13.

### Fabrication processes

The 4 two-input NAND gate chip was fabricated based on 0.2 ${\upmu }$m P-well SPDM CMOS processes, with the minimum feature size being 0.2 ${\upmu }$m. To improve the radiation-resistance of the chip, the chips were rapidly treated in a thermal ammonia atmosphere at 950 °C for 30 seconds. As a result, the interface states, fixed positive charges, and dangling bonds in the $Si-SiO_2$ interface region were considerably reduced, and the bond strength was also enhanced.

### Measurement results

The measured main PCM, DC and dynamic parameters of chips were listed in Tables 3, 4 and 5, respectively.

**Table 3:** Measured PCM.

Items	Sample 1	Sample 2	Sample 3	Sample 4	Sample 5	Requirement	Unit
N_$V_t$	0.886	0.891	0.898	0.903	0.894	$0.9 \pm 0.15$	V
N_$BV_d$	13.48	13.56	13.44	13.4	13.64	$> 10$	V
P_$V_t$	− 0.977	− 0.971	− 0.971	− 0.967	− 0.972	$-1.0 \pm 0.15$	V
P_$BV_d$	− 14.72	− 14.72	− 14.64	− 14.72	− 14.72	$< -10$	V
R_$N^+$	55.621	55.892	56.216	55.491	58.318	$50 \pm 20$	$\Omega /\text {sq}$
R_$P^+$	88.222	89.144	90.199	88.780	90.822	$80 \pm 20$	$\Omega /\text {sq}$
R_*Poly*	25.065	25.283	25.588	24.875	25.837	$25 \pm 5$	$\Omega /\text {sq}$
R_$N^+$ con	32.835	33.071	34.276	33.001	33.555	$< 150$	$\Omega /\text {cont}$
R_$P^+$ con	67.422	68.844	67.606	68.559	70.537	$< 150$	$\Omega /\text {cont}$
N_$V_{tf}$	13.84	14.16	14.44	13.88	14.12	$> 12$	V
P_$V_{tf}$	− 17.96	− 17.92	− 17.76	− 18.12	− 17.96	$< -12$	V
R_$P-Well$	4270.74	4260.75	4279.37	4272.33	4275.2	$3500 \pm 500$	$\Omega /\text {sq}$

**Table 4:** Measured DC parameters.

Para.	Testing conditions			Typical value	Specification value		unit	Qualified
		$V_{CC}$	$T_A$		Min.	Max.		
Output high level voltage $V_{OH}$	$I_{OH}$ = − 20 ${\upmu }$A	2.0 V	-55 $^{\circ }$C	2.001529–2.001837	1.9	/	V	$\checkmark$
			25 $^{\circ }$C	2.006414	1.9			
			125 $^{\circ }$C	1.999527	1.9			
		4.5 V	-55 $^{\circ }$C	4.501933–4.502405	4.4			
			25 $^{\circ }$C	4.511623	4.4			
			125 $^{\circ }$C	4.500516–4.501461	4.4			
		6.0 V	-55 $^{\circ }$C	6.005751–6.006696	5.9			
			25 $^{\circ }$C	6.00613–6.011014	5.9			
			125 $^{\circ }$C	6.004806–6.005278	5.9			
	$I_{OH}$ = − 4 mA	4.5 V	-55 $^{\circ }$C	4.285568–4.301739	3.7			
			25 $^{\circ }$C	4.25016–4.260143	3.98			
			125 $^{\circ }$C	4.162827–4.179305	3.7			
	$I_{OH}$ = − 5.2 mA	6.0V	-55 $^{\circ }$C	5.778973–5.796454	5.2			
			25 $^{\circ }$C	5.732625–5.747277	5.48			
			125 $^{\circ }$C	5.656608–5.672671	5.2			
Output low level voltage $V_{OL}$	$I_{OL}$ = 20 ${\upmu }$A	2.0 V	-55 $^{\circ }$C	0.002018–0.002205	/	0.1	V	$\checkmark$
			25 $^{\circ }$C	0.002266		0.1		
			125 $^{\circ }$C	0.00305–0.003237		0.1		
		4.5 V	-55 $^{\circ }$C	0.001142–0.001299		0.1		
			25 $^{\circ }$C	− 0.000229–0.002266		0.1		
			125 $^{\circ }$C	0.001674–0.001924		0.1		
		6.0 V	-55 $^{\circ }$C	0.001205–0.001549		0.1		
			25 $^{\circ }$C	− 0.000229–0.002266		0.1		
			125 $^{\circ }$C	0.001643–0.002143		0.1		
	$I_{OL}$ = 4 mA	4.5 V	-55 $^{\circ }$C	0.134451–0.144208		0.4		
			25 $^{\circ }$C	0.174478–0.184462		0.26		
			125 $^{\circ }$C	0.238335–0.248216		0.4		
	$I_{OL}$ = 5.2 mA	6.0 V	-55 $^{\circ }$C	0.144396–0.154653		0.4		
			25 $^{\circ }$C	0.184462–0.194445		0.26		
			125 $^{\circ }$C	0.001736–0.259411		0.4		
Input current $I_{I}$	$V_{I}$ = $V_{CC}$ or 0	6.0 V	-55 $^{\circ }$C	0.009858–0.044062		±1.0	${\upmu }$A	$\checkmark$
			25 $^{\circ }$C	− 0.014953–0.022414		±0.1		
			125 $^{\circ }$C	0.00563–0.046367		±1.0		
Power supply current $I_{CC}$	$V_{I}$ = $V_{CC}$ or 0 $I_O$ = 0	6.0 V	-55 $^{\circ }$C	− 0.000718–0.012285		40	${\upmu }$A	$\checkmark$
			25 $^{\circ }$C	0.00082–0.010946		2		
			125 $^{\circ }$C	− 0.074541–0.033566		40

**Table 5:** Measured dynamic parameters ($T_A$ = 25 $^{\circ }$C).

Parameters	$V_{CC}$	Typical values	Specification value		Unit	Qualified
			Min.	Max.		
Transmission delay time $t_{PLH}$	2.0 V	38.0–39.8	/	90	ns	$\checkmark$
	4.5 V	11.8–13.3		18		
	6.0 V	8.7–10.8		15		
Transmission delay time $t_{PHL}$	2.0 V	42.1–43.9	/	90	ns	$\checkmark$
	4.5 V	12.7–13.7		18		
	6.0 V	9.7–10.9		15		
Allowable time $t_{THL}$	2.0 V	14.7–15.1	/	75	ns	$\checkmark$
	4.5 V	7.7–8.1		15		
	6.0 V	4.8–5.1		13		
Allowable time $t_{TLH}$	2.0 V	14.7–15.0	2.5	12	ns	$\checkmark$
	4.5 V	7.7–8.1				
	6.0 V	4.8–5.1				

It can be seen from table3, 4 and 5 that the practically tested PCM, DC and dynamic parameters are much superior to the corresponding specification values. It is indicated that circuits, layouts and technological process parameters were designed correctly and properly.

## Conclusions

The 4 two-input NAND gate was designed and fabricated based on 5 V, 1.2 ${\upmu }$m P-well SPDM CMOS processes, using a single metal layer and N-well P-substrate CMOS with only one polycrystalline layer. The designed and fabricated device with 4 two-input NAND gate has many excellent advantages, such as low static power consumption, high input impedance, strong anti-interference capability, large load capability, and high noise tolerance. The maximum input current is only 11 ${\upmu }$A, and the maximum power supply current is 40 ${\upmu }$A. It has a strong output driving capability and can drive at least 10 LSTTL loads. The N-transistors were fabricated in P-well and connected to GND via $P^+$-well. The metal wires of $V_{CC}$ use $N^+$ substrate contact $V_{CC}$ to increase its anti-latching capability. The performances of the designed device are superior to those of other similar products in the world. The comparisons of the performance parameters of the fabricated device with optimum specifications in the world are given in Table 6.

**Table 6:** Comparisons with international specifications.

Parameter	Sign	Testing conditions		Testing results	International specifications		Unit
					lower limit	upper limit	
Output high level voltage	$V_{OH}$	$I_{O}$ = − 20 ${\upmu }$A	$V_{CC}$ = 2 V	2.003918–2.006414	1.9	/	V
			$V_{CC}$ = 4.5 V	4.252656–4.511623	4.4	/	
			$V_{CC}$ = 6 V	5.9993–5.9995	5.9	/	
		$I_{O}$ = − 4 mA, $V_{CC}$ = 4.5 V		4.25016–4.511623	3.98	/	
		$I_{O}$ = − 5.2 mA, $V_{CC}$ = 6 V		6.00613–6.011014	5.48	/	
Output low level voltage	$V_{OL}$	$I_{O}$ = 20 ${\upmu }$A	$V_{CC}$ = 2 V	0.002266	/	0.1	V
			$V_{CC}$ = 4.5 V	− 0.000229–0.002266	/	0.1	
			$V_{CC}$ = 6 V	− 0.000229–0.002266	/	0.1	
		$I_{O}$ = 4 mA, $V_{CC}$ = 4.5 V		0.174478–0.184462	/	0.26	
		$I_{O}$ = 5.2 mA, $V_{CC}$ = 6 V		0.184462–0.194445	/	0.26	
Input current	$I_{I}$	$V_{CC}$ = 6 V		− 0.014953–0.022414	/	±0.1	${\upmu }$A
Power supply current	$I_{CC}$	$V_{CC}$ = 6 V		0.00082–0.010946	/	2	${\upmu }$A
Transmission delay time	$t_{PHL}$, $t_{PLH}$	$t_{r}$ = 6 ns $t_{f}$ = 6 ns $C_{L}$ = 50 pF	$V_{CC}$ = 2 V	38.0–43.9	/	90	ns
			$V_{CC}$ = 4.5 V	11.8–13.7	/	18	
			$V_{CC}$ = 6 V	8.7–10.9	/	15	
Conversion time	$t_{THL}$, $t_{TLH}$	$t_{r}$ = 6 ns $t_{f}$ = 6 ns $C_{L}$ = 50 pF	$V_{CC}$ = 2V	14.7–15.1	/	75	ns
			$V_{CC}$ = 4.5 V	7.7–8.1	/	15	
			$V_{CC}$ = 6 V	4.8−5.1	/	13	

## Data Availability

All data generated or analysed during this study are included in this published article.

## References

[1] Swaroop, B. S., Saxena, A. & Sahay, S. Satisfiability attack-resilient camouflaged multiple multivariable logic-in-memory exploiting 3d nand flash array. *IEEE Trans. Circuits Syst. I: Regul. Pap.* **71**, 660–669. 10.1109/TCSI.2023.3326332 (2024).

[2] Yee, O. & King, L. Nano-mosfets implementation of different logic families of two inputs nand gate transistor level circuits A simulation study. *J. Teknol. (Sci. Eng.)* **79**, 41–49. 10.11113/jt.v79.9947 (2017).

[3] Suh, D. I., Kil, J. P., Kim, K. W., Kim, K. S. & Park, W. A single magnetic tunnel junction representing the basic logic functions-nand, nor, and imp. *IEEE Electron Device Lett.* **36**, 402–404. 10.1109/LED.2015.2406881 (2015).

[4] Kumar, M., Hussain, M. & Paul, S. Performance of a two input nand gate using subthreshold leakage control techniques. *J. Electron Devices.* **14**, 1161–1169 (2020).

[5] Vardhan, B. V., Khadir, M., Sunnyhith, K. & Paruchuri, S. Design and implementation of low power nand gate based combinational circuits using finfet technique. In *2023 14th International Conference on Computing Communication and Networking Technologies (ICCCNT)* (2023).

[6] Canan, T. F., Kaya, S., Karanth, A. & Louri, A. 4-input nand and nor gates based on two ambipolar schottky barrier finfets. In *2020 27th IEEE International Conference on Electronics, Circuits and Systems (ICECS)*, 1–4, 10.1109/ICECS49266.2020.9294892 (2020).

[7] Channi, M. K. & Kumar, M. Design and evaluation of low power 2 to 4 decoder circuit using three and four transistors nand gates. In *2024 International Conference on Integrated Circuits, Communication, and Computing Systems (ICIC3S)*, vol. 1, 1–5, 10.1109/ICIC3S61846.2024.10602861 (2024).

[8] Lee, T. *Ultra low power CMOS medium frequency power collection and communication circuits for remote sensor nodes*. Ph.D. thesis, University of Southampton (2019).

[9] Lee, S.-T., Woo, S. Y. & Lee, J.-H. Low-power binary neuron circuit with adjustable threshold for binary neural networks using nand flash memory. *IEEE Access* **8**, 153334–153340. 10.1109/ACCESS.2020.3018226 (2020).

[10] Muthu, K. E., Firthouse, V. J. U., Deepa, S. S., Raja, A. S. & Robinson, S. Design and analysis of 3-input nand/nor/xnor gate based on 2d photonic crystals. *J. Opt. Commun.* **43**, 181–189. 10.1515/joc-2018-0210 (2022).

[11] Son, J., Shin, Y., Cho, K. & Kim, S. Logic-in-memory operation of ternary nand/nor universal logic gates using double-gated feedback field-effect transistors. *Adv. Electron. Mater.* **9**, 2201134. 10.1002/aelm.202201134 (2023).

[12] Kotb, A., Zoiros, K. E. & Li, W. Realization of ultrafast all-optical NAND and XNOR logic functions using carrier reservoir semiconductor optical amplifiers. *J. Supercomputing* **77**, 14617–14629. 10.1007/s11227-021-03876-4 (2021).

[13] Zhao, Z., Zhang, Y., Wang, P., Zhang, H. & Weishan, Z. Design of crosstalk nand gate circuit based on interconnect coupling capacitance. In *2019 IEEE 13th International Conference on ASIC (ASICON)*, 1–4, 10.1109/ASICON47005.2019.8983533 (2019).

[14] Lee, S.-T. & Lee, J.-H. Review of neuromorphic computing based on nand flash memory. *Nanoscale Horiz.* **9**, 1475–1492. 10.1039/D3NH00532A (2024). 39015048 10.1039/d3nh00532a

[15] Zhu, Y. et al. Monolithic dual-gate e-mode device-based nand logic block for gan mis-hemts ic platform. *IEEE J. Elect. Devices Soc.* **11**, 230–234. 10.1109/JEDS.2023.3265372 (2023).

